# Proximity-ligation metagenomics reveals disease-specific mobilome dynamics in disrupted gut ecosystems

**DOI:** 10.21203/rs.3.rs-9142184/v1

**Published:** 2026-03-25

**Authors:** Andrew J Sommer, Benjamin Auch, Alexander Khoruts, Jasmohan S Bajaj

**Affiliations:** 1 Department of Medicine, Division of Gastroenterology, University of Minnesota, Minneapolis, MN, USA; 2 Phase Genomics, Seattle, WA, USA; 3 Center for Immunology, University of Minnesota, Minneapolis, MN USA; 4 Department of Medicine, Division of Gastroenterology, Hepatology, and Nutrition, Virginia Commonwealth University and Richmond VA Medical Center, Richmond, VA, USA

## Abstract

Distinct ecological pressures shape accumulation of antimicrobial resistance and virulence genes in the gut microbiome. Using proximity ligation shotgun metagenomics to resolve host–mobilome relationships, we analyzed microbiomes from two patient cohorts: recurrent *Clostridioides difficile* infection (rCDI) and cirrhosis. While rCDI reflects antibiotic-driven disruption, cirrhosis-driven microbiome changes result from altered gut physiology. We found increased chromosomal determinants of antibiotic resistance in both, but plasmid-mediated amplification was more evident in rCDI.

The human gut microbiome plays a central role in host physiology and colonization resistance against pathogens^1^, yet it also represents a major reservoir of antimicrobial resistance genes (ARGs) and virulence factors (VFs)^2^. High bacterial density and spatial organization within the gut create favorable conditions for horizontal gene transfer (HGT). These processes allow resistance and virulence traits to disseminate rapidly across taxa and contribute to the emergence of multidrug-resistant opportunistic pathogens^2,3^.

Members of the Enterobacteriaceae and Enterococcaceae families are of particular concern because they frequently cause bloodstream, intra-abdominal and urinary tract infections in medically complex patients. The surveillance of plasmid-borne extended spectrum β-lactamases (ESBLs) and vancomycin resistance genes (VRGs) within pathogenic lineages is of urgent clinical relevance; however, conventional shotgun metagenomics cannot reliably assign mobile genetic elements or ARGs to bacterial hosts^4^. Proximity ligation shotgun metagenomics (PLSM), also referred to as Hi-C metagenomics, addresses this limitation by cross-linking DNA molecules that are co-localized within intact cells prior to sequencing, allowing plasmids and bacteriophages to be assigned directly to host genomes and enables direct reconstruction of host-mobilome interactions^5,6^

We applied PLSM to fecal microbiomes from healthy individuals and two disease states associated with contrasting disrupted gut ecosystems: recurrent *Clostridioides difficile* infection (rCDI) and cirrhosis. rCDI microbiomes experience prolonged antibiotic exposure and inflammatory perturbations, whereas cirrhosis is characterized by liver and immune dysfunction, altered gut physiology, and impaired barrier integrity, typically before extensive antibiotic use^7–9^. Comparing these cohorts therefore provides an opportunity to distinguish antibiotic-driven from physiologically-driven influences on mobilome evolution.

We analyzed 66 stool samples [34 rCDI, 19 cirrhosis (5 compensated/14 decompensated) and 13 healthy, extended tables/text]. rCDI patients had prolonged antibiotic exposure for recurrent infection (6.9 ± 3.9 months). In contrast, we only included cirrhosis patients without absorbable antibiotic exposure, although half of decompensated patients received rifaximin for hepatic encephalopathy (HE) prophylaxis. PLSM generated an average of ~61 million reads per sample and enabled assembly of 2,070 bacterial metagenome-assembled genomes (MAGs) with ≥70% completeness and ≤5% contamination.

Healthy individuals exhibited microbiomes dominated by obligate anaerobic Clostridia, including Lachnospirales and Oscillospirales (extended Data Fig.1). In contrast, both rCDI and decompensated cirrhosis microbiomes showed increased abundance of Enterobacteriaceae and Enterococcaceae. Community disruption was most pronounced in rCDI samples, which also contained MAGs absent from healthy individuals, including Veillonellaceae and Fusobacteriaceae. Cirrhosis samples displayed a less pronounced shift toward Enterobacteriaceae, with a visual gradient between compensated and decompensated disease. These findings suggest that host physiological changes alone can create ecological conditions favoring facultative anaerobes.

Across all samples we assembled 705 plasmids and 2,521 bacteriophages with ≥50% completeness and ≤5% contamination. Although total plasmid abundance did not differ significantly between cohorts, plasmid host associations differed markedly (extended Data Fig.2). In healthy individuals, plasmids were primarily linked to Bacteroidaceae. In contrast, both rCDI and cirrhosis microbiomes exhibited increased plasmid associations with Enterobacteriaceae. Enterobacteriaceae-associated plasmids were significantly enriched in rCDI compared with both cirrhosis and healthy cohorts, whereas Bacteroides-associated plasmids declined. Thus, rCDI was characterized not simply by increased plasmid abundance but by a shift toward plasmids carried by clinically high-risk taxa. Notably, substantial inter-individual variability was observed, indicating that mobilome composition can differ markedly even within similar clinical contexts.

Enterobacteriaceae carried the largest ARG burden across all cohorts (Fig.1a). Both rCDI and cirrhosis microbiomes exhibited increased chromosomally encoded ARGs relative to healthy controls, consistent with expansion of Enterobacteriaceae taxa harboring intrinsic resistance determinants such as efflux pumps and β-lactamases (Fig.1b). In contrast, plasmid-borne ARGs showed a striking disease-specific pattern. Plasmid-encoded β-lactamases, including *blaTEM-1, blaOXA-1, blaSHV-27 and blaCTX-M*, were detected in rCDI microbiomes and were absent from cirrhosis and healthy samples despite cirrhosis-associated Enterobacteriaceae expansion and rifaximin use (Fig.1c extended data Fig.3). These findings suggest that intense antibiotic exposure in rCDI creates ecological conditions that favor plasmid-mediated horizontal gene transfer and maintenance of resistance elements, whereas physiological disruption in cirrhosis without absorbable antibiotics does not drive widespread plasmid-mediated ARG amplification.

ARG and VF abundance within Enterobacteriaceae showed a strong correlation (ρ = 0.81), driven largely by *Escherichia* MAGs (Fig.1d and extended data Fig.4). In rCDI samples, MAGs encoding plasmid-borne β-lactamases frequently co-encoded plasmid-borne siderophore systems, adhesins and colicin genes characteristic of extraintestinal pathogenic *E. coli* (ExPEC) (Fig.2a)^10,11^. In contrast, *Klebsiella* MAGs encoding plasmid β-lactamases rarely carried additional acquired virulence determinants, possibly reflecting the importance of variable capsular polysaccharide and lipopolysaccharide loci in the *K. pneumoniae* core genome^12^. These observations suggest that *E. coli* may act as a key mobilome hub linking resistance and virulence traits within gut reservoirs.

While VRGs were absent from healthy microbiomes, *Enterococcus* MAGs encoding VRGs were identified in several rCDI and cirrhosis patients (Fig.2b and extended data Fig.5). A single rCDI sample also contained a *C. difficile* MAG carrying VanG-type resistance genes, potentially enabling survival during ongoing vancomycin therapy. VRGs were also detected in cirrhosis patients without prior vancomycin exposure, reflecting a shift towards gram-positive taxa changes in cirrhosis and suggesting that ecological disruption alone may permit colonization by resistant *Enterococcus* populations^13^.

Despite enrichment of ARGs and VFs in disease cohorts, we observed no consistent relationship between resistance burden and conventional healthcare exposure metrics such as antibiotic duration, care intensity, or patient demographics (extended data Fig.6 and Fig.7). Some rCDI patients with modest healthcare exposure carried high ARG/VF loads, whereas others with extensive treatment histories did not. Similarly, rifaximin use, which has been linked to increased resistance to the last-resort antibiotic daptomycin^14^, was not significantly associated with increased Enterobacteriaceae ARG or VF abundance in cirrhosis patients (extended data Fig.7). When we compared the 10 rifaximin users (3 rCDI+7 decompensated cirrhosis) to non-users (decompensated and rCDI), we found no statistical differences in Enterobacteriaceae VF, Enterobacteriaceae ARG, and VRG abundance across samples (extended data Fig.8). These findings indicate that accumulation of resistance and virulence genes reflects complex interactions among microbial community structure, host physiology, environmental exposure, and mobile genetic element dynamics rather than simple antibiotic burden alone.

Limitations of this study include modest sizes of patient cohorts, clinical presentations potentially reflecting center-specific practices, enrolling of cirrhosis patients with minimal systemic antibiotic exposure, and the sequencing of a single fecal sample time point per patient. Nonetheless these findings demonstrate that distinct ecological pressures shape mobilome evolution in disrupted gut microbiomes. rCDI microbiomes, subjected to sustained antibiotic exposure, exhibit strong plasmid-mediated amplification of resistance genes and coupling of resistance and virulence traits within *E. coli*. In contrast, cirrhosis microbiomes accumulate genomic ARGs despite relatively limited absorbable antibiotics exposure, indicating that host-driven ecological disruption can prime microbiomes for resistance acquisition.

By resolving host–mobile element relationships at scale, PLSM provides a powerful tool for studying the evolutionary processes that drive the emergence of resistant and virulent pathobionts within the human microbiome. These results from two distinct but common conditions, rCDI and cirrhosis, both associated with disrupted gut ecosystems, highlight the importance of ecological context in shaping resistance reservoirs and underscore the need for microbiome-preserving therapeutic strategies.

## Supplementary Material

Supplementary Files

This is a list of supplementary files associated with this preprint. Click to download.

• ExtendedDataMethods31626.docx

• Extendeddataresultstext31626.docx

• Extended gures31626labeled.pdf

• ExtendedDataTables31626.xlsx

## Figures and Tables

**Fig.1:**
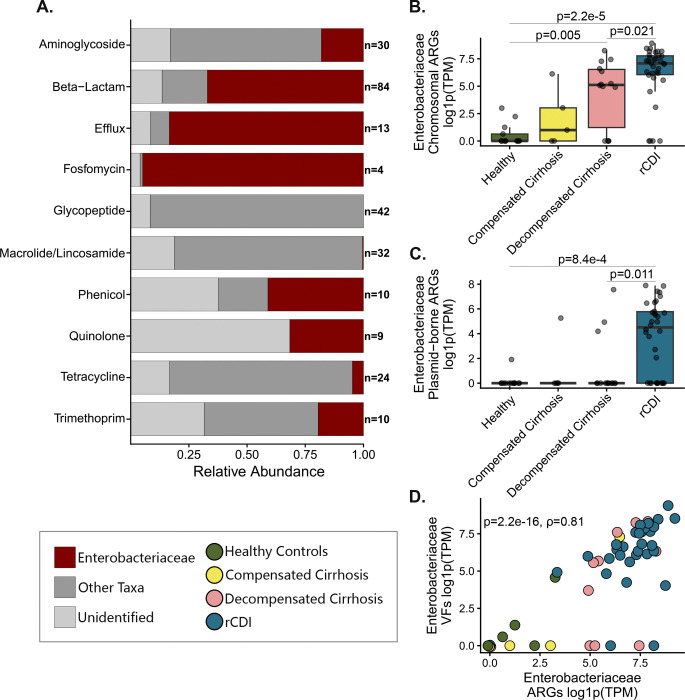
Distribution and abundance of Enterobacteriaceae ARGs and VFs. **a**, Relative abundance of ARG classes encoded by Enterobacteriaceae. **b,c,** TPM-normalized abundance of chromosomally encoded (**b)** and plasmid-encoded (**c**) Enterobacteriaceae ARGs across sample types. **d,** Scatterplot showing the relationship between ARG and VF abundance across samples.

**Fig.2:**
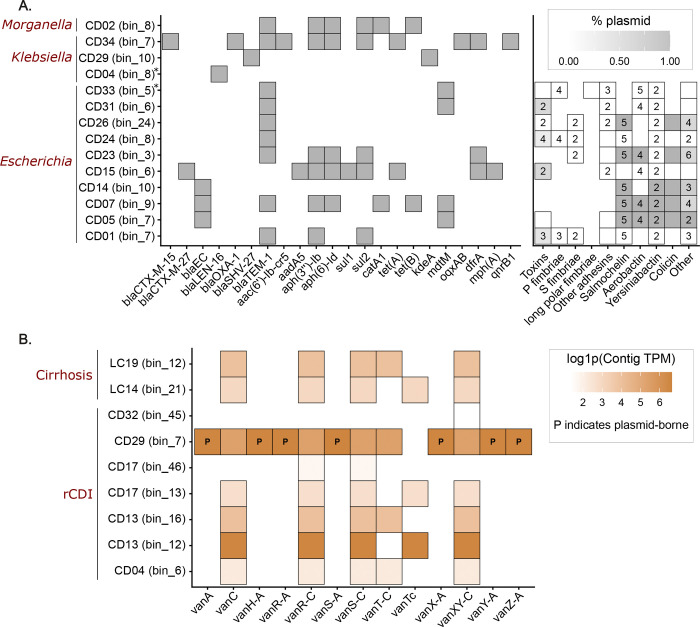
High Risk Enterobacteriaceae and *Enterococcus* MAGs identified through PLSM. **a,** VF distribution in Enterobacteriaceae MAGs encoding plasmid-borne β-lactamase genes in rCDI samples. Boxes without a number represent a single gene identified within the functional category. Asterisk indicates Klebsiella strains not classified as *K. pneumoniae* or *Escherichia* strains not classified as *E. coli*. **b,** Distribution of *Enterococcus* MAGs encoding VRGs. Color intensity reflects the contig-level abundance of the VRG.

## Data Availability

Raw metagenomic shotgun sequencing reads are pending submission to the NCBI Sequence Read Archive (SRA) under BioProject PRJNA1438274. All analysis code is available as a GitHub repository: https://github.com/asommer101-dot/2026-PLSM-Enterobiome [Released Upon Acceptance] All methods and tables are available as extended data.
